# HtrA1L364P leads to cognitive dysfunction and vascular destruction through TGF‐β/Smad signaling pathway in CARASIL model mice

**DOI:** 10.1002/brb3.2691

**Published:** 2022-07-15

**Authors:** Li Chuanfen, Wang Xiaoling, Jing Wen, Cao Bingzhen, Wang Min

**Affiliations:** ^1^ Shandong Normal University, College of Physical Education Sports Human Science Laboratory Jinan Shandong China; ^2^ Neurology Department PLA 960th Hospital Jinan Shandong China

**Keywords:** CARASIL, Mut‐HtrA1L364P mice, pathogenic mechanism, phenotypic analysis

## Abstract

**Aims:**

Cerebral autosomal recessive arteriopathy with subcortical infarcts and leukoencephalopathy (CARASIL) is a life‐threatening, inherited, nonhypertensive arteriole disease of the brain. Therapeutic strategy for CARASIL is limited because its pathogenesis is not clear. We previously reported the first family with CARASIL in China, which involves a high‐temperature requirement serine protease gene mutation (HtrA1^L364P^). Based on this previous study, we constructed a CARASIL mouse model (Mut‐hHtrA1^L364P^ mouse, hereinafter referred to as Mut). This paper aimed to systematically study the behavior, pathology, and molecular biology of Mut mice and explore the pathogenesis and possible therapeutic strategies of CARASIL.

**Methods:**

Food maze and water maze experiments were used in the behavioral studies. Pathological studies were carried out by arteriole labeling staining and electron microscopy. The mRNA and protein expression levels of the key factors of TGF‐β/Smad signaling pathway (TGF‐β, Smad2, Smad3, and Smad4) in the brain of the model mice were detected by immunohistochemistry, real‐time quantitative polymerase chain reaction (RT‐PCR), and Western blot assay.

**Results:**

The food maze and water maze experiment data showed significant differences between the Mut and wild‐type (WT) mice in the first time to find food, the time to contact the escape table for the first time, and the number of times to travel in the escape table quadrant (*p* < 0.001). The results of vascular labeling staining showed that some small arteries in the brain of Mut mice lost normal structure. The results of electron microscopy showed that the cell morphologies in the cortex and hippocampus of Mut mice were abnormal; the number of synapses was reduced; the walls of capillaries, venules, and arterioles thickened; lumen stenosis and other abnormal phenomenon occurred; and lipofuscin deposition and autophagosomes were found in the hippocampus. Immunohistochemistry, RT‐PCR, and Western Blot results showed that the mRNA and protein expression levels of TGF‐β, Smad2, and Smad3 in the brain of Mut mice increased to different degrees.

**Conclusions:**

The most significant innovation of this study is the first study on the pathogenesis of CARASIL disease using model animals. The Mut mice can well simulate the pathogenesis of CARASIL in behavioral and pathological aspects. The TGF‐β/Smad signaling pathway, which is involved in the pathogenesis of CARASIL, is abnormally upregulated in the brain of Mut mice.

## Graphical abstract

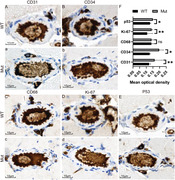

## 1 INTRODUCTION

Cerebral autosomal recessive arteriopathy with subcortical infarcts and leukoencephalopathy (CARASIL) is a nonhypertensive genetic cerebral vascular disease, which was first reported by Japanese scientists in 1960 (Uemura et al. 2020). The disease has a low incidence but a high fatality rate, and its unique clinical manifestations include early‐onset dementia in young people, stroke, low back pain, and baldness (Ziaei et al. 2019). Brain magnetic resonance shows diffuse white matter lesions, subcortical infarction, concentric vascular wall thickening, lumen narrowing, intimal fibrous hyperplasia, and massive loss of smooth muscle cells in the brain similar to arteriosclerosis (Liu et al. 2020). (Hara et al. 2009) found that CARASIL is related to high‐temperature requirement serine peptidase A1 (HtrA1). In 2011, Fukutake (2011) confirmed that the disease is an autosomal recessive inheritance through the genetic separation ratio analysis of 10 families with blood marriage from Japan and located the pathogenic gene in HtrA1. Patients mostly die of small cerebral artery infarction within 10 years after the onset of neurological symptoms (Badachi et al. 2020). The pathogenesis of this disease has not been clarified; thus, the disease has no effective treatment program, and the clinical interventions only relieve the disease symptoms. Therefore, an in‐depth investigation of its pathogenesis is of great importance for guiding the precise clinical treatment of this disease.

Our research team reported the first Chinese family with CARASIL in 2012, and the clinical symptoms all met the diagnostic criteria of CARASIL (Wang et al. 2012). Sequencing results showed that T1091C mutation occurred in the cDNA of HtrA1 and resulted in the mutation of leucine (Leu, L) at site 364 to proline (Pro, P). We transfected the HtrA1^L364P^ mutant gene into human vascular smooth muscle cells. The mutant gene decreased the proliferation and migration, increased the apoptosis, and activated the transforming growth factor‐β/Smad (TGF‐β/Smad) signaling pathway in the cells. We successfully constructed a mouse model that expresses the human mutant gene HtrA1^L364P^ using the CRISPR/Cas9 system to further investigate the pathogenesis of CARASIL (Li et al. 2019). HtrA1 is a highly conserved serine protease that, at normal physiological concentrations, inhibits the TGF‐β/Smad signaling pathway, which plays an important role in the regulation of vascular integrity (Oluwole et al. 2020). Previous in vitro studies have shown that mutations in the *HtrA1* gene result in loss of the HtrA1 protein and decreased protease activity (Tossetta et al. (2016). This mutation results in the abnormal elevation of the TGF‐β signaling pathway in cerebral arterioles, as well as the accumulation of TGF‐β members and TGF‐β signal‐inducing proteins (Lin et al. 2018). (Li et al. 2020). In addition, the acceleration of the TGF‐β signaling pathway may lead to the widespread loss of vascular smooth muscle cells in patients with CARASIL (Zurawa‐Janicka et al. 2017). Therefore, the upregulation of the TGF‐β signaling pathway is suggested to be involved in the pathogenesis of CARASIL (Moreno‐Garcia et al. 2018). We hypothesized that *HtrA1* ^L364P^ gene mutation in CARASIL mouse model leads to cognitive dysfunction and vascular structural damage through the TGF‐β/Smad signaling pathway.

In this study, we conducted a systematic phenotypic analysis and a pathological study on transgenic mice (herein called Mut mice) and analyzed the pathogenic mechanism further. The key factors of the TGF‐β/Smad signaling pathway were selected as the research targets to study the influence of HtrA1^L364P^ mutation on this signaling pathway. The role of changes in this signaling pathway in the occurrence and development of CARASIL was also explored.

## 2 ANIMALS AND METHODS

### 2.1 Animals

All animal experiments were approved by the Animal Experimental Ethics Committee of Shandong Normal University. C57BL/6J mice were purchased from Model Animal Research Center of Nanjing University (Nanjing, China). The animals were maintained in a standard light–dark cycle with an ambient temperature of 22 ± 4°C. The mice had ad libitum access to water and food. All mice were acclimatized for a week without any experimentation. The Mut mice were transgenic mice constructed in the early stage of our study.

*Animal groups*: The control group was composed of wild‐type (WT) C57BL/6J mice, and the model group was consisted of Mut mice. The number of mice used in the experiment was 90.

### 2.2 Food Y maze experiment

Fifteen male 8‐month‐old WT and Mut mice were selected. The mice were fasted and only given water 24 h before the experiment. The experiment consisted of two phases with 1 h apart. The first stage is the training period, and the second stage is the detection phase. Food was randomly placed on one end of an arm of the Y maze, and the mice were placed on the starting arm. The video was recorded and timed. The time from the starting arm to finding the food was recorded, and the number of times (this number was defined as the number of errors) that the mice entered another arm besides the starting arm and the feeding arm before finding the food was recorded. The experiment was done once a week for 5 weeks, followed by a day of fasting with water treatment. The first 4 weeks were the training stage, and the fifth week was the experimental test. Each mouse was tested three times, and the data were recorded and analyzed in detail.

### 2.3 Water maze experiment

The groups for the water maze experiment are the same as those in Section 2.2. The water maze test includes two parts: positioning and navigation training and space exploration test. Each mouse was tested four times a day for 4 consecutive days. The mice were randomly placed in a different starting point each time. The interval between each test was 6 min. During the test, the mice were placed into the water facing the wall of the pool and allowed to swim free for 120 s. The time of finding the platform (ESCAPE Latency, EL), the residence time in the quadrant of the platform, movement distance, and movement trajectory were recorded. Then, space exploration test (spatial probe test) was conducted as follows. The underwater platform is removed 24 h after the last training test (the fifth day), and then the mice were placed back in. SuperMaze video analysis system was used to keep track of mice activities within 2 min to analyze the residence time and the number of shuttles in the escape platform.

### 2.4 Pathological experiment: cerebral arteriole labeling test

Fifteen 8‐month‐old WT and Mut mice were randomly selected respectively. This part included routine hematoxylin and eosin (HE) staining, elastic fiber staining, specific immunohistochemical staining (smooth muscle actin [SMA], actin, CD31, CD34, CD68, Ki‐67, and P53 staining), and light microscope observation. The brain tissues of male WT mice and Mut mice were fixed with paraffin wax and then sliced into 5 μm sections. All sections were deparaffinized, rehydrated, and stained through standard protocol. The sections were then photographed and examined. The product number and concentration of the antibodies used in the immunohistochemical experiment were as follows: SMA (Abcam 5694; 1:200), actin (Abcam 8227; 1:200), CD31 (Abcam 182981; 1:200), CD34 (Abcam 8158; 1:200), CD68 (Abcam 125212; 1:200), Ki‐67 (Abcam 92742; 1:200), P53 (Abcam 245740; 1:200). The secondary antibody kit is a general two‐step detection kit (Jinqiao PV‐9000; Beijing, China).

### 2.5 Electron microscopy

The mice were grouped in the same way as above. The mice were infused with 4% paraformaldehyde for 30 min and then dissected. Brain tissues were crosscut. Small tissue pieces were collected from the cerebral cortex and hippocampus and cut into 1 × 1 × 2 mm^3^ tissue blocks with blades. The tissue blocks were quickly placed in 2.5% glutaraldehyde fixation solution for fixation at room temperature for 2 h and then fixed overnight at 4°C for electron microscopy specimen preparation. Afterward, the samples were washed, fixed with 1% osmium acid for 1.5 h, and embedded with EPON 812 resin after gradient dehydration. The samples were cut into ultrathin sections with 70 nm thickness, stained with 2% uranium dioxide acetate and lead citrate, and observed by electron microscopy.

### 2.6 Immunohistochemistry

The groups, brain samples, and tissue specimen processing for immunohistochemistry are the same as those in Section 2.4. The expression differences of key factor proteins in the TGF‐β/Smad signaling pathway were detected. The primary antibodies were mouse monoclonal antibodies against TGF‐β (Abcam 215715; 1:200), Smad2 (Abcam 40855; 1:200), Smad3 (Abcam 40854; 1:200), and Smad4 (Abcam 40759; 1:200). The results were observed with an optical microscope and photographed. First, the levels of TGF‐β, Smad2, Smad3, and Smad4 in the two groups of tissues were qualitatively analyzed by integral comprehensive metering method, and then the average optical density (accumulated optical density value/accumulated area) was quantitatively analyzed by the image processing software, ImageJ.

### 2.7 Real‐time polymerase chain reaction

The mRNA expression levels of key factors in the TGF‐β/Smad signaling pathway were detected by real‐time polymerase chain reaction (RT‐PCR) assay. Total RNA was extracted using an ultra‐pure RNA extraction kit (Tiangen, Beijing), and mRNA was reverse transcribed into cDNA using a kit for RT‐PCR (Tiangen, Beijing). The primer sequences are shown in Table 1. The Ct indexes of the WT and Mut mice were separately subtracted from the internal gene Ct value to obtain the ΔCt value. Then, 2^−ΔΔCt^ was used to calculate the relative expression levels of HtrA1^L364P^ in WT and Mut mouse cells.

**TABLE 1:** RT‐PCR primer sequence

Gene	5′−3′
TGF‐β	ATGGTGGACCGCAACAACGC
	GGCACTGCTTCCCGAATGTCTG
Smad2	CCGTGCTCCCTCCGTCTTCC
	CTGCCGCCCGCTGATTGG
Smad3	TTGACAGAGAGCAACACAGTAT
	CTTCATCCAGATCGATTGCTTG
Smad	GTTGCCTGAAGCCTGGAAGTGG
	TCCTGCCGTCTGTTGAATGTGC
β‐actin	TGACGTGGACATCCGCAAAG
	CTGGAAGGTGCACAGAGAGG

### 2.8 Western blot

The protein extracted from the brain samples were resolved equally by sodium dodecyl sulfate–polyacrylamide gel electrophoresis and subsequently transferred onto Immobilon‐P membranes (Millipore, Shanghai, China). Precision Plus protein standards (2 mg; Bio‐Rad Laboratories, Hercules, CA, USA) were loaded into the first lane of the gel. Then, the membranes were incubated in 5% nonfat milk at room temperature for 1 h and then incubated with primary antibodies in 5% nonfat milk at 4°C overnight. Afterward, the membranes were exposed to secondary antibodies. The signals of the proteins of interest were then detected using the SuperSignal West Femto Maximum Sensitivity Substrate Kit (Thermo Scientific, Shanghai, China) according to the manufacturer's instructions. The antibodies used in this experiment were the same as above.

### 2.9 Data processing and analysis

SPSS 17.0 statistical software was used to analyze the experimental data, and the results are expressed as mean ± standard deviation. Independent sample *t* test was used in the statistical analysis of the results between the two groups in animal experiments. Excel 2010 was used for data processing and image generation. *** represents a very significant difference between the two groups (*p* < .001), ** represents a significant difference between the two groups (*p* < .01), and * represents a comparison difference between the two groups (*p* < .05), ns represents no difference between the two groups.

## 3 RESULTS

### 3.1 Mut mice had decreased cognitive function

**FIGURE 1:**
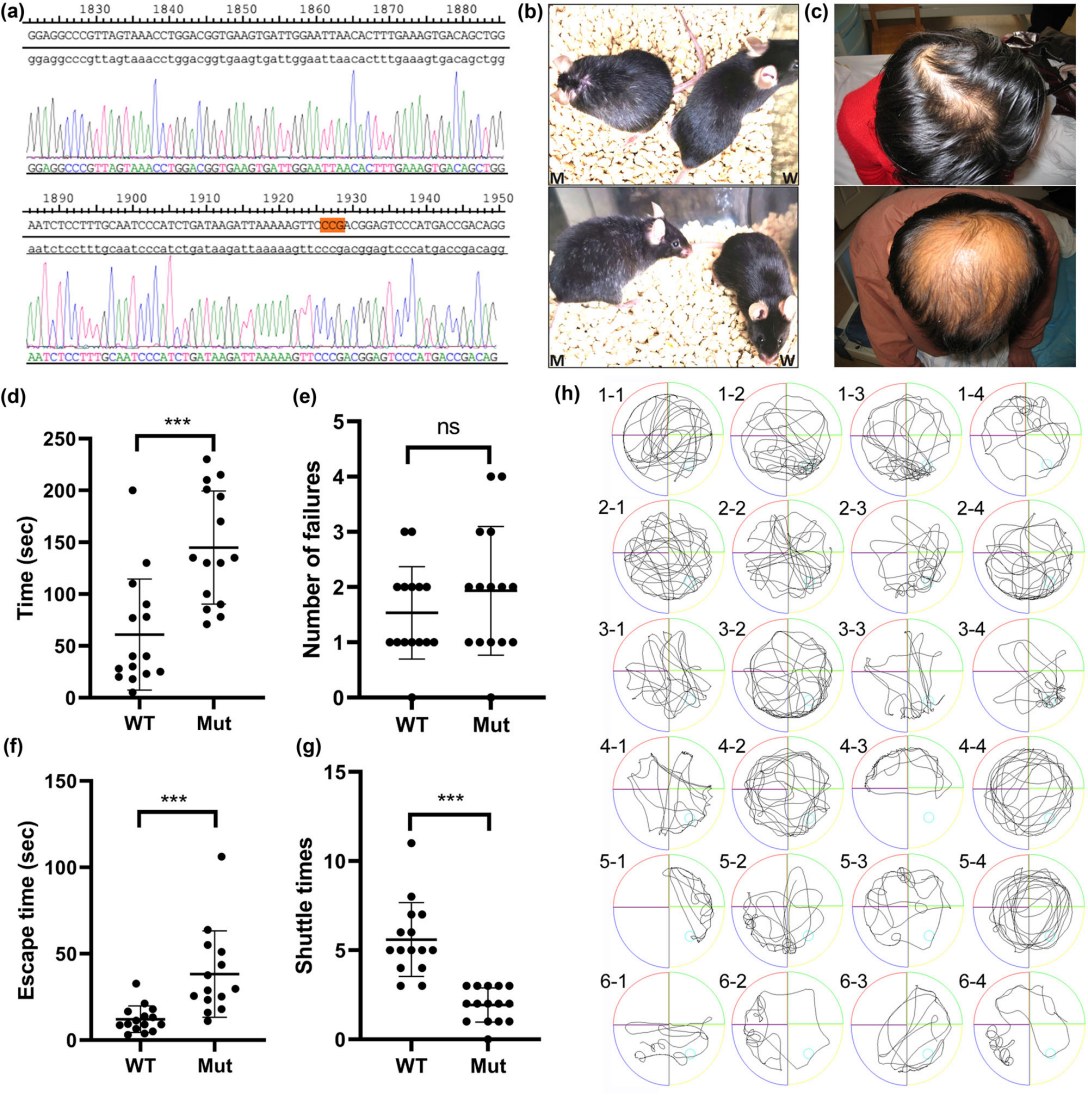
Gene identification map, phenotypic analysis and behavioral analysis of Mut mice. (a) Gene identification map of homozygous mut mice. The red mark is the mutant target gene. (b) Hair map of mut mice. M represents mut mice and W represents WT mice. (c) Hair pictures of clinical patients. The two patients were siblings. (d) Time required for mice to find food in the Y maze. (e) Number of errors in Y maze food search. (f) Time required for mice to contact the escape platform for the first time. (g) Number of times the mice shuttle in the escape platform area. (h) 1, 2, and 3 are WT mice; 4, 5, and 6 are Mut mice. 1‐1 represents the movement trajectory of the water maze device in 120 s after the first quadrant of the first mouse, and so on. ns (*p* > .05), ***(*p* < .001) compared with the control group

Figure 1(a) is the gene identification map of homozygous mut mice. The red mark is the mutant target gene. Figure 1(b) is the hair map of mut mice. Figure 1(c) is the hair pictures of clinical patients. The two patients were siblings. As shown in Figures 1(b) and 1(c), the model mice and patients have similar hair sparsity, which is an important basis for the clinical diagnosis of CARASIL patients.

In Food Y maze experiment, 15 mice were chosen from each group. The average time to find food for the first time for Mut mice was more time (144.93 ± 54.63 s) than that of WT mice (60.93 ± 53.60 s, *p* < .001), but the average number of errors was not significantly different between Mut mice (1.93 ± 1.16 times) and WT mice WT (1.53 ± 0.83 times, *p* > .05).

In water maze experiment, 15 mice were chosen from each group, too. The average time to contact the escape platform for the first time for Mut mice was more time (44.02 ± 25.05 s) than that of WT mice (11.57 ± 8.50 s, *p* < .001), and the average number of shuttles in the escape platform area was very significantly different between Mut mice (1.75 ± 0.97 times) and WT mice WT (5.83 ± 2.25 times, *p* < .001).

The experimental data of food maze and water maze are analyzed, and the results are shown in Figures 1(d), 1(e), 1(f), and 1(g). Figure 1(h) shows the action trajectory map of some experimental mice in the water maze experiment.

### 3.2 Mut mice had disordered vascular structure in the brain and remarkably thickened vascular walls

#### 3.2.1 Vascular labeling staining

**FIGURE 2:**
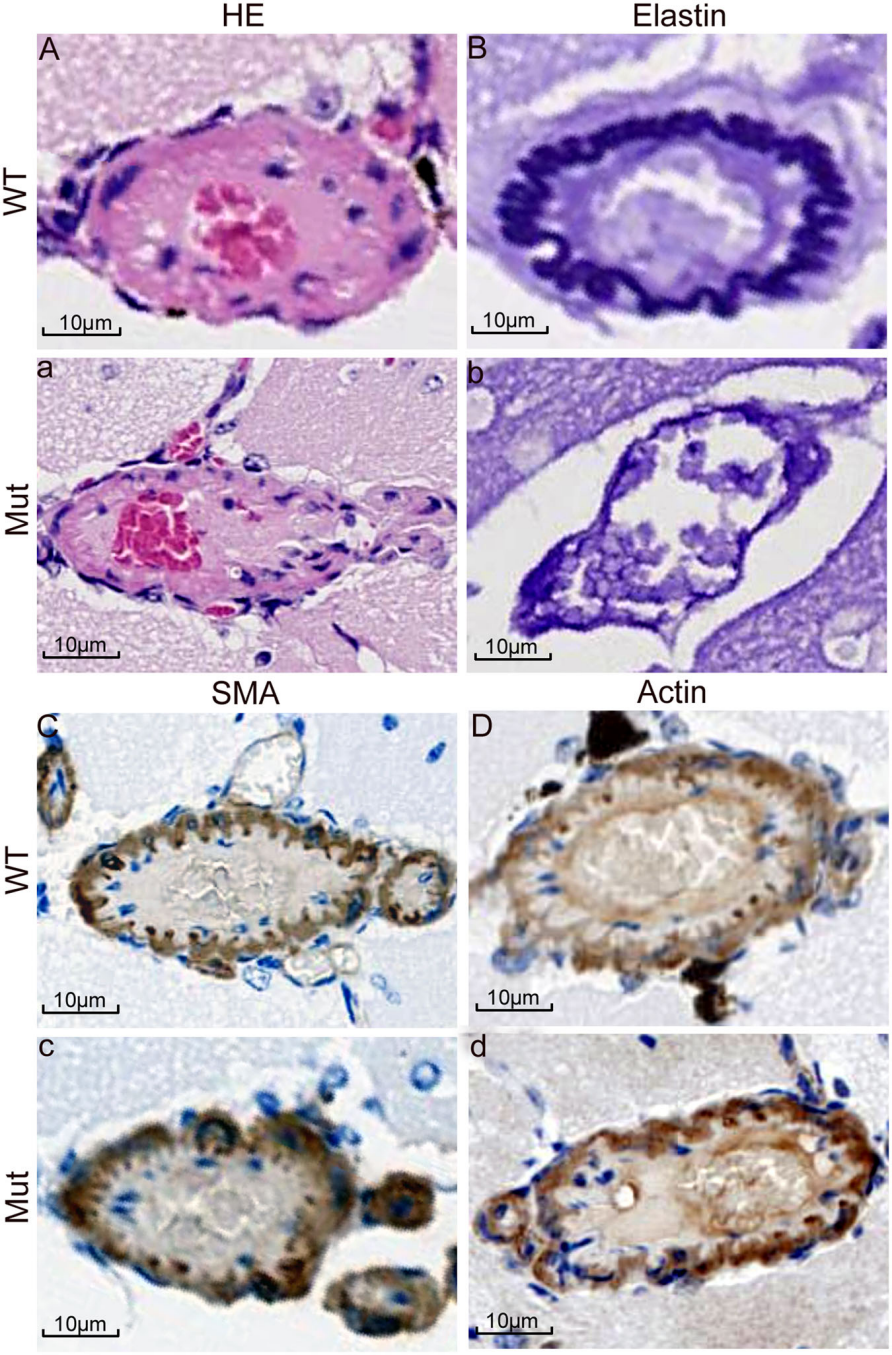
Small arterioles in the brain were labeled and stained. Capital letters A–D are pathological staining of the brain of WT mice, respectively: HE, elastin, actin, SMA. Lowercase letters a–d represent the staining corresponding to the Mut group, respectively. The calipers represent 10 μm

**FIGURE 3:**
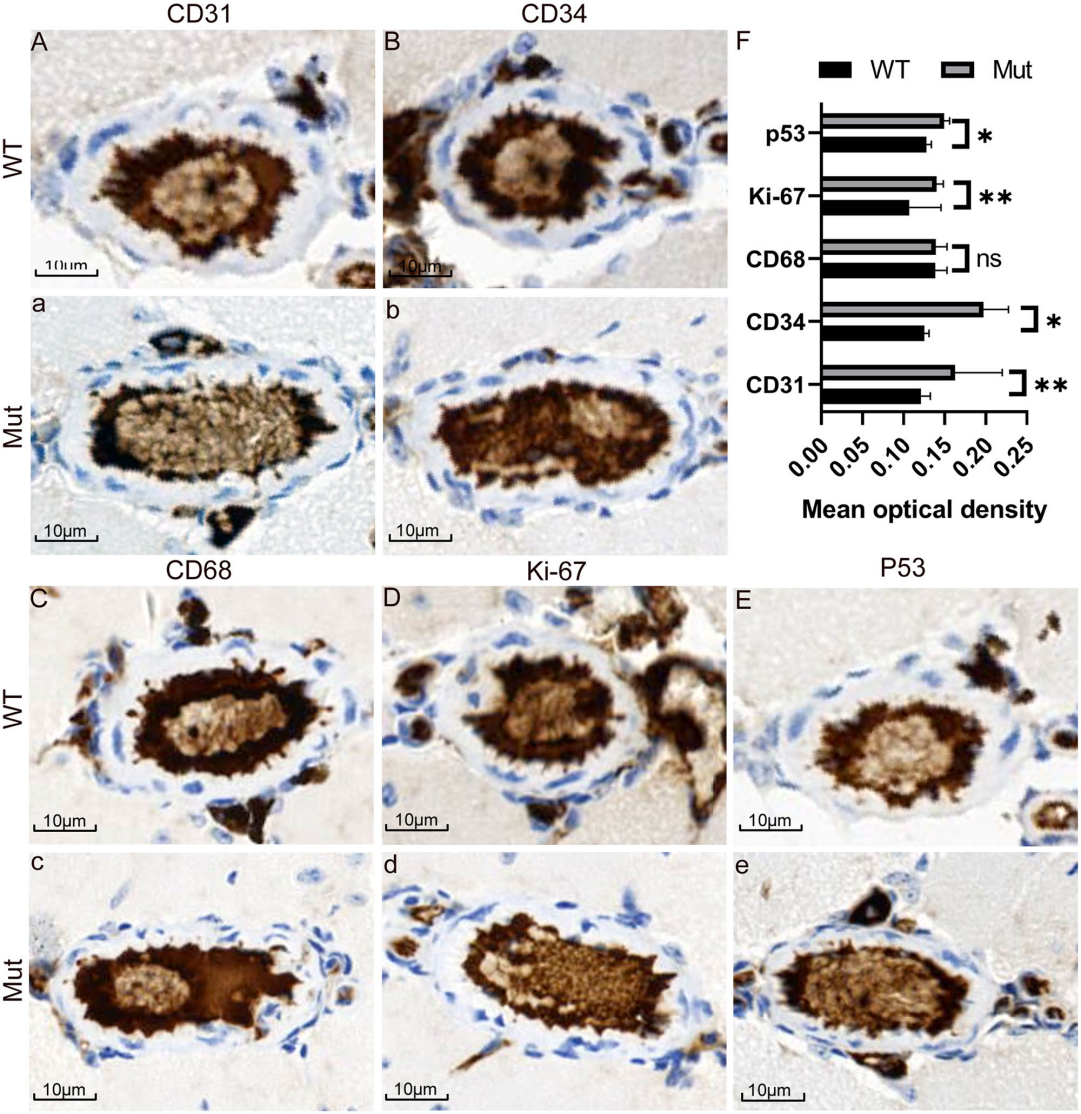
Small arterioles in the brain were labeled, stained, and quantitative analysis. Capital letters A–E are pathological staining of the brain of WT mice, respectively: CD31, CD34, CD68, Ki‐67, and p53. Lowercase letters a–e represent the staining corresponding to the Mut group, respectively. f is the optical density quantitative analysis of two groups of immunohistochemical staining. The calipers represent 10 μm

We conducted vascular marker staining experiments to study the small arterial structure of the Mut mouse brain. As shown in Figures 2 and 3, the small arteries in the brain vessels of the 15 Mut mice have different degrees of damage, including vascular wall hyperplasia, structural disorder, and lumen stenosis.

Brain staining results showed that the vascular elastic fiber structure of WT mice was orderly and complete. HE, elastin, actin, and SMA staining showed that the vascular wall structure was complete and tightly arranged. The endothelial cells labeled by CD31 and CD34 antibodies and the macrophages labeled by CD68 antibody were orderly and tightly arranged. Compared with WT mice, Mut mice showed disordered elastic fiber structure, disordered actin and SMA arrangements, thinned vascular wall, narrowed lumen, and the loss of the normal arrangement of CD31 and CD34 antibody‐labeled endothelial cells and CD68 antibody‐labeled macrophages. The results of optical density analysis of immunohistochemistry showed that CD31, CD34, Ki‐67, and p53 in the vascular wall of cerebral arterioles of the model mice had abnormal proliferation in varying degrees, and there was no difference in the expression of CD68 between the two groups.

#### 3.2.2 Electron microscopy

**FIGURE 4:**
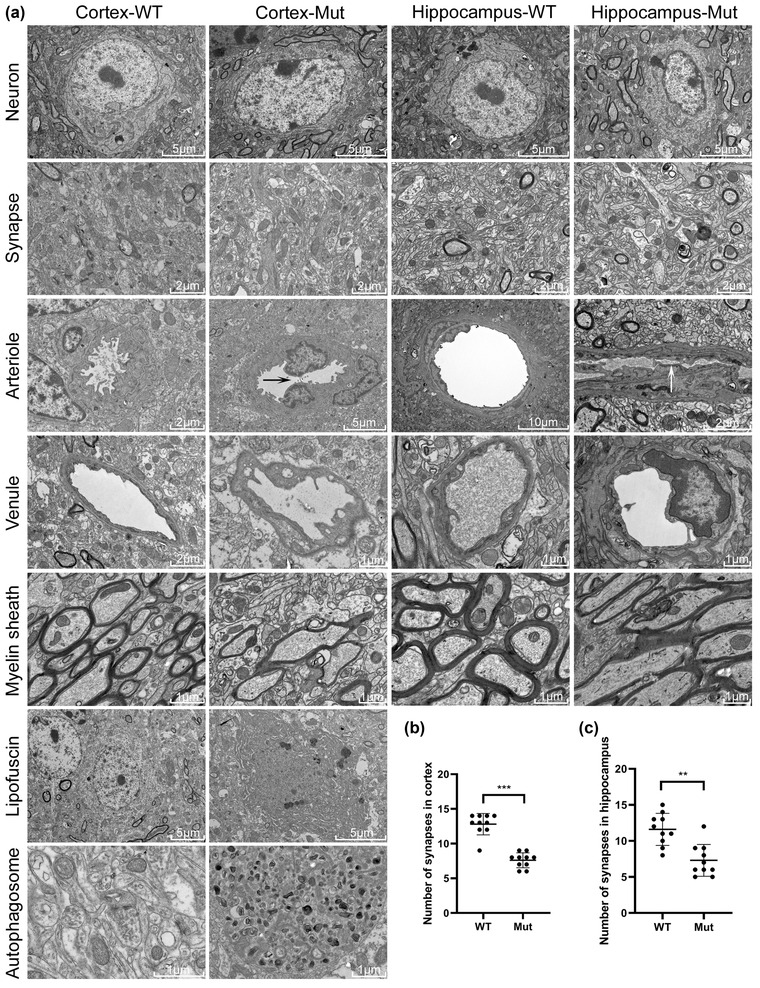
Electron microscopic observation of the cerebral cortex and hippocampus. (a) Electron microscopy results of the cerebral cortex and hippocampus of the two groups of mice. Neurons, synapses, arterioles, venules, and myelin sheaths are shown in the cerebral cortex and hippocampus of the two groups of mice. Abnormal lipofuscin and autophagosomes were observed in cortical sites. (b and c) Statistical analyses of synapses in the cortex and hippocampus of the two groups of mice. Many aging neurons and abnormal subcellular structures were found in the brains of Mut mice. The number of synapses was remarkably reduced. Some capillaries, venules, and arterioles were abnormal in shape. Abnormal endothelial cell proliferation, mitochondria; swelling in endothelial cells, lumen narrowing, lipofuscin accumulation, and abnormal autophagosomes in some cells were observed. The myelin structure of the WT mice was intact, whereas that of the Mut mice was abnormal with varying degrees of disintegration, degeneration, and swelling in the myelin lamella. The number of synapses in Mut mice was significantly lower than that in WT mice (*p* < .001). The red asterisk denotes the synapse.

Figure 4 shows the cortical and hippocampus tissues of the two groups of mice under electron microscopy. The small arteries and venules of the brain of mice in the Mut group had abnormal vascular structure. The medial membrane of the aorta wall thinned, and the endothelium proliferative vascular lumen narrowed. The narrowest point is less than 2 μm (as shown by the white and black arrow). In addition, abnormal phenomena, such as vascular wall swelling and structural disorder, were found in the venules. Furthermore, the results show the presence of many aging neurons and abnormal subcellular structures in the brains of Mut mice, and the number of synapses was remarkably reduced. The specific accumulation of a large number of lipofuscin and abnormal autophagosomes were found in many cells of the brain cortex. The myelin structure of the cerebral cortex and hippocampus of the two groups of mice were observed under electron microscopy. The results showed that the myelin structures of the cerebral cortex and hippocampus of WT mice were intact, whereas those in Mut mice was abnormal with varying degrees of disintegration, degeneration, and swelling in the myelin lamella.

The number of synapses was calculated randomly under electron microscopy and statistically analyzed by independent sample *t*‐test. The results showed that the number of synapses in Mut mice was substantially lower than that in WT mice. The statistical analysis results in the cortex and hippocampus are shown in Figures 4(b) and 4(c), respectively.

### 3.3 The TGF‐β/Smad signaling pathway was activated in the brain of the mice

**FIGURE 5:**
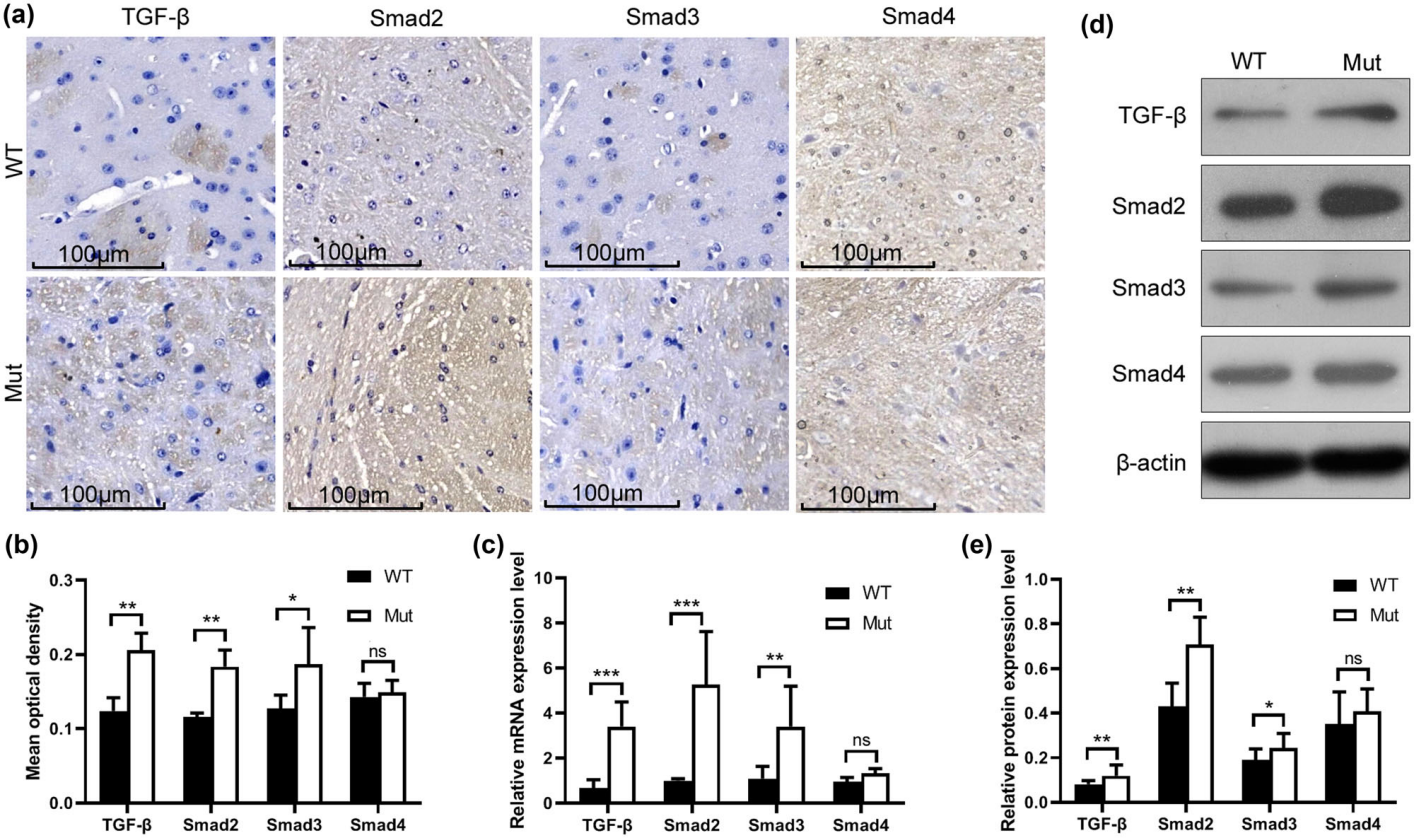
Detection of TGF‐β/Smad signaling pathway in the brain of the mice. (a) Immunohistochemistry images of TGF‐β, Smad2, Smad3, and Smad4 expression in paraffin sections of the brain tissues of WT and Mut mice. (b) Average optical density of the immunohistochemical images analyzed by ImageJ software. Black and white represent the relative protein expression levels of TGF‐β, Smad2, Smad3, and Smad4 in the brains of WT and Mut mice, respectively. The expression levels of TGF‐β and Smad2 between the two groups were significantly different (*p* < .01), the expression of Smad3 was different between the two groups (*p* < .05), and the expression of Smad4 was not different between the two groups (*p* > .05). (c) Compared with WT mice, the mRNA expression levels of TGF‐β in the brain of Mut mice extremely significantly increased (*p* < .001), the mRNA expression levels of Smad2 and Smad3 significantly increased (*p* < .01), and the mRNA expression levels of Smad4 were not significantly different between the two groups (*p* > .05). (d) The protein expression levels of TGF‐β and Smad2 in Mut mice significantly increased compared with those in WT mice (*p* < .01), the protein expression level of Smad3 increased compared with the control group (*p* < .05), and no significant difference was observed in the protein expression level of Smad4 between the two groups (*p* > .05)

The expression levels of the key factors of the TGF‐β/Smad signaling pathway (TGF‐β, Smad2, Smad3, and Smad4) in WT and Mut mice were detected by immunohistochemistry (Figure 5(a)).

The results of immunohistochemistry were analyzed by integral comprehensive measurement method. We randomly selected 10 fields under a 40× optical microscope and counted the positive staining cells (+ represents the total number of positive cells less than 25%, ++ represents the total number of positive cells between 25 and 49%, and +++ represents more than 50% of the total number of positive cells). The degree of coloration score (0 is negative coloration, 1 is light yellow, 2 is light brown, and 3 is dark brown) was used in integral comprehensive measurement. The formula used was: (+)%×1 +(++)%×2+(+++)%×3. Total value of <1.0, 1−1.5, and >1.5 indicated (+), (++), and (+++), respectively.

The results of the integral comprehensive measurement showed that the expression levels of TGF‐β, Smad2, and Smad3 in Mut mice increased in varying degrees compared with those in WT mice, whereas Smad4 showed no substantial difference between the two groups (Table 2).

**TABLE 2:** Comprehensive measurement results of immunohistochemical integral

	Group	
Antigen	WT	Mut
TGF‐β	++	+++
Smad2	+	++
Smad3	++	+++
Smad4	++	++

Then, the immunohistochemistry results were quantitatively analyzed by ImageJ software. The results are similar to those of the integral synthesis method (Figure 5(b)). The expression levels of TGF‐β and Smad2 were significantly different between the two groups (*p* < .01), the expression of Smad3 was different between the two groups (*p* < .05), and the expression of Smad4 was not different between the two groups (*p* > .05).

RT‐PCR was used to detect the mRNA expression differences of the key factors in the TGF‐β/Smad signaling pathway. Total RNA is complete without degradation and thus can be used for subsequent quantitative PCR analysis, and cDNA samples were amplified by fluorescence quantitative PCR (SYBR Green). The analysis of the melting curve showed that the products amplified by each sample were all unimodal, which indicates that each target gene underwent specific amplification.

The RT‐PCR results (Figure 5(c)) showed that compared with WT mice, the mRNA expression levels of TGF‐β in the brain of Mut mice were extremely significantly increased (*p* < .001), the mRNA expression levels of Smad2 and Smad3 were significantly increased (*p* < .01), and the mRNA expression levels of Smad4 were not significantly different between the two groups (*p* > .05).

Western blot results (Figures 5(d) and 5(e)) showed that the protein expression levels of TGF‐β and Smad2 in Mut mice significantly increased compared with those in WT mice (*p *< .01). The protein expression level of Smad3 increased compared with the control group (*p* < .05), whereas the protein expression level of Smad4 was not significantly different between the two groups (*p* > .05).

## 4 DISCUSSION

CARASIL is a rare form of inherited arteriole disease (Uemura et al. 2020). It is a genetic disease caused by HtrA1 gene mutation (Zhang and Yang 2020). By 2020, 27 CARASIL families have been identified by genetic testing (20 inbred families), and 22 *HtrA1* mutation sites were found; among which, 20 were homozygous mutations, two were compound heterozygous mutations, and one was heterozygous mutation (Hou et al. 2020). The *HtrA1* gene has nine exons. The reported missense mutations were located in the protease active region of exons 3, 4, and 6 of HtrA1: 496C>T(R206C) (Souza et al. 2016), 616G>A(A206G) (Ibrahimi et al. 2017), 754G>A(A252T), 889G>A(V297M), 883G>A(G295A) (Mendioroz et al. 2010), 1091T>C (L364P), 854C>T(P285L) (Chen et al. 2013), and 821G>A(A274G) (Yu et al. 2020). Although the disease‐causing genes have been well understood, the mechanism of the disease has not been revealed due to the lack of a typical animal model of the disease. Based on the mutated gene from a clinical family [1091 T>C (L364P)], we successfully constructed a model mouse for CARASIL.

The biggest innovation of this study is the first systematic study of the disease using CARASIL model mice. We comprehensively analyzed the CARASIL mouse model (MUT htra1l364p) from the perspectives of behavior, pathology, and molecular biology. First of all, the Mut mice generally showed phenotypes such as less hair and more white hair than WT of the same month, which was consistent with alopecia and less hair, one of the typical symptoms of clinical patients (Figures 1(b) and 1(c)). Second, in the food maze and water maze experiments, after strict and identical training, the average time to find food for the first time and the average time to find the lifeguard for the first time, Mut mice were significantly higher than WT mice (Figures 1(d) and 1(f)), and the shuttle times in the area where the lifeguard was located were significantly lower than WT mice (Figure 1(g)), indicating that their learning and memory ability and spatial positioning ability were significantly lower. There was no significant difference in the number of errors between the two groups in the food maze experiment (Figure 1(e)), which may be due to the slow action of Mut mice.

According to the above experimental data, Mut mice have obvious learning, memory and cognitive impairment. What is the reason for this phenomenon? On clinical patients, head MRI or CT often shows extensive subcortical white matter demyelination and multiple cerebral infarction. Brain pathology in autopsy cases revealed arteriolar atherosclerosis, including severe hyaline degeneration of the media, massive loss of smooth muscle cells, thickened intimal fibrosis, thickened and broken internal elastic layer, and centripetal stenosis of the lumen (Uemura et al. 2017). (Arima et al. 2003). On this basis, we conducted a blood vessel labeling experiment on the brain tissue of mut mice. The results showed that the arrangement of elastic fibers, actin and SMA in Mut mice was disordered, the vascular wall was significantly thinner, the lumen narrowed, the arrangement of endothelial cells and macrophages also lost normal order, the intimal hyperplasia of small artery wall was abnormal, and the proliferative activity of intimal cells was increased (Figures 2 and 3). This indicates that the cerebral arterioles of Mut mice are damaged.

In order to further verify the degree of brain injury, we then carried out electron microscopic examination of brain tissue (Figure 4). Interestingly, the electron microscopy results showed that the synapses in the cortex and hippocampus of Mut mice were remarkably reduced (Figures 4(b) and 4(c)). The number, density, and plasticity of synapses are positively correlated with the learning and memory abilities of the body and the ability to adapt to the environment (Scholl et al. 2021), which explains the poor learning and memory abilities of Mut mice. In addition, the myelin structure of the cerebral cortex and hippocampus of Mut mice had different degrees of abnormalities, including degeneration, disintegration, swelling, cavity, and axonal injury, as observed under electron microscopy. The myelin sheath contains many lipids and can act as an insulator; therefore, the electrical impulses of nerve fibers can be transmitted quickly and have good insulation performance. Lesions in the myelin sheath are bound to affect nerve conduction velocity, and the symptoms include mental symptoms, such as memory loss, arthroscopy, handicapped limb movements, and paralysis (Liu et al. 2019). Pathological and electron microscopy results showed that Mut mice had vascular endothelial cell hyperplasia, thickened intima, narrow lumen, disordered structure, and no amyloid deposition. These findings are consistent with previous reports, but the pathogenesis needs to be further investigated. Another surprising finding was that large amounts of lipofuscin deposits were found in the cerebral cortex of Mut mice. Lipofuscin deposition in nerves can lead to impaired endothelial cell function in vascular wall and promote atherosclerosis (Nishimoto et al. 2011). Therefore, whether lipofuscin is one of the culprits in CARASIL disease needs to be further studied.

So far, we have determined that the brain injury of Mut mice leads to the impairment of brain function. So, what is the relationship between HtrA1 gene mutation and brain injury? What mechanism causes brain injury after gene mutation? Most of the target proteins of HtrA1 are in the extracellular matrix (ECM), and the activity of serine protease expressed by HtrA1 is closely related to the TGF‐β signaling pathway and can inhibit the expression of this signaling pathway (Yamashiro et al. 2020). The TGF‐β signaling pathway affects the generation of ECM by regulating downstream molecules (Xiong et al. 2019). The relationship between HtrA1 gene mutation and cerebrovascular pathological damage in CARASIL is also a focus in the study of the TGF‐β signaling pathway. Our results showed that the TGF‐β signaling pathway was indeed abnormally activated in Mut mice (Figure 5). Previous studies demonstrated that mutated HtrA1 gene leads to reduced serine protease activity, which is unable to inhibit the TGF‐β signaling pathway; the overexpression of this pathway promotes the deposition of ECM (Yokobori and Nishiyama 2017). Immunohistochemical studies also confirmed the deposition of some ECM components on the cerebrovascular wall in CARASIL (Oide et al. 2008). Takehiko speculated that the excessive deposition of ECM further leads to the proliferation of vascular intima, the division of the inner elastic layer, and vascular smooth muscle degeneration and thus subsequently leads to cerebral arteriolar stenosis in CARASIL and the occurrence of clinical neurological symptoms and the corresponding extensive white matter lesions found in imaging (Yokobori and Nishiama 2017). Lumbar lesions and hair loss are related to bone morphogenetic protein metabolism, which is a member of the TGF‐β family (Chen et al. 2018). HtrA1 and TGF‐β are widely expressed in a variety of tissues and cells in the body, but why does *HtrA1* gene mutation selectively affect small intracranial arteries? HtrA1 and TGF‐β are speculated to be important to maintain the structural integrity of small intracranial arteries. TGF‐β is expressed in astrocytes, microglia, endothelial cells, and vascular smooth muscle cells in the central nervous system, and these cells are the major components of the neurovascular unit (NVU) in the central nervous system. NVU plays an important role in maintaining the structural and functional integrity of intracranial microvascular and nervous tissue (Parkes et al. 2018). (Niu et al. 2017b). Thus, the role of the TGF‐β signaling pathway mediated by *HtrA1* gene mutation on NVU deserves further study.

### PEER REVIEW

The peer review history for this article is available at https://publons.com/publon/10.1002/brb3.2691.
